# Recent Innovations of Mesoporous Silica Nanoparticles Combined with Photodynamic Therapy for Improving Cancer Treatment

**DOI:** 10.3390/pharmaceutics16010014

**Published:** 2023-12-20

**Authors:** Doaa Sayed Nady, Afnan Hassan, Muhammad Umair Amin, Udo Bakowsky, Sherif Ashraf Fahmy

**Affiliations:** 1 Department of Chemistry, Faculty of Science, Cairo University, Giza 12613, Egypt; 2 Biomedical Sciences Program, Zewail City of Science and Technology, Giza 12578, Egypt; 3 Department of Pharmaceutics and Biopharmaceutics, University of Marburg, Robert-Koch-Str. 4, 35037 Marburg, Germany; 4 Department of Chemistry, School of Life and Medical Sciences, University of Hertfordshire Hosted by Global Academic Foundation, R5 New Garden City, New Capital, Cairo 11835, Egypt

**Keywords:** photodynamic therapy, photosensitizers, nanocarriers, silica nanoparticles, mesoporous silica nanoparticles, cancer

## Abstract

Cancer is a global health burden and is one of the leading causes of death. Photodynamic therapy (PDT) is considered an alternative approach to conventional cancer treatment. PDT utilizes a light-sensitive compound, photosensitizers (PSs), light irradiation, and molecular oxygen (O_2_). This generates cytotoxic reactive oxygen species (ROS), which can trigger necrosis and/ or apoptosis, leading to cancer cell death in the intended tissues. Classical photosensitizers impose limitations that hinder their clinical applications, such as long-term skin photosensitivity, hydrophobic nature, nonspecific targeting, and toxic cumulative effects. Thus, nanotechnology emerged as an unorthodox solution for improving the hydrophilicity and targeting efficiency of PSs. Among nanocarriers, mesoporous silica nanoparticles (MSNs) have gained increasing attention due to their high surface area, defined pore size and structure, ease of surface modification, stable aqueous dispersions, good biocompatibility, and optical transparency, which are vital for PDT. The advancement of integrated MSNs/PDT has led to an inspiring multimodal nanosystem for effectively treating malignancies. This review gives an overview of the main components and mechanisms of the PDT process, the effect of PDT on tumor cells, and the most recent studies that reported the benefits of incorporating PSs into silica nanoparticles and integration with PDT against different cancer cells.

## 1. Introduction

In recent years, cancer has been one of the leading causes of death globally, and it continues to be a primary public health burden [1]. The term cancer refers to a group of medical conditions originating from abnormal regulation and cell growth that lead to unregulated proliferation and neoplastic development coupled with metastatic ability [2,3]. In 2020, more than 19.2 million new cancer cases were diagnosed, and around 9.9 million patients died worldwide [4]. This number is predicted to rise by 70% by 2040 [5]. Many studies have reported that alcohol, advanced age, smoking, genetic mutations, hormonal imbalances, and poor lifestyle can contribute to cancer development [2,5]. Nowadays, different types of clinical treatments for cancer are being used, such as radiotherapy, chemotherapy, and surgery. However, these conventional treatments still show some drawbacks, such as low curative effects, poor selectivity, painful treatment procedures, toxic effects on normal cells [6,7], and multidrug resistance [8,9,10]. In this context, it becomes essential to develop alternative safe treatment processes that are more effective, facile, and less invasive [6].

**Figure 1:**
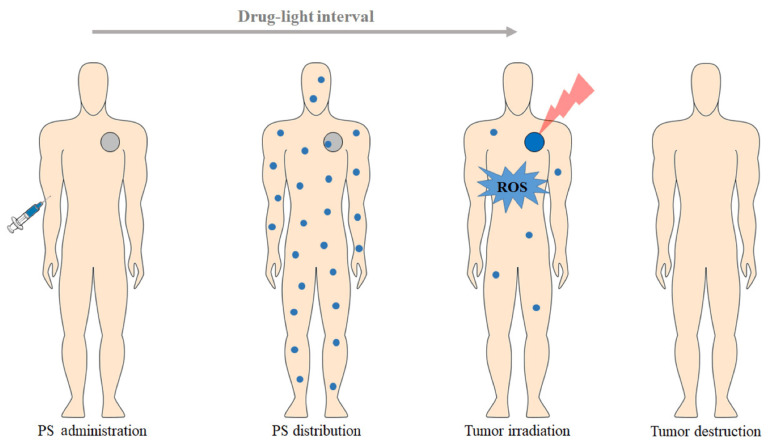
A schematic overview representing the clinical application of photodynamic therapy protocol for cancer treatment [13].

Photodynamic therapy (PDT) has gained much attention in the past decades for treating various kinds of cancer, such as lung, skin, bladder, esophageal cancer, etc. [1,2,11]. Photodynamic therapy is a modern, rapidly developing, and noninvasive method for diagnosing and treating cancer disease [12]. PDT involves using a photosensitizer (PS) that is administered locally or systemically with a nontoxic dose. Initially, the PS is nonspecifically distributed throughout the body, accumulating in the tumor tissues. The typical duration for the PS to reach its target site is reported to be between 5 min and 24 h, depending on the method of administration and type of photosensitizers. Then, the target tissue is exposed to light irradiation with a specific wavelength called the photodynamic window (600–850 nm), resulting in the formation of cytotoxic reactive oxygen species (ROS) in the presence of endogenous molecular oxygen (O_2_), leading to the eradication or regression of cancer cells (Figure 1) [4,12].

Photodynamic therapy has attracted significant attention, mainly in cancer treatment, owing to its spatial and temporal selectivity, where irradiation can be controlled in terms of time and position. Additionally, it is well tolerated by patients as it can be applied at the same site more than once. Moreover, it preserves fertility and does not affect pregnancy and delivery [14,15]. Other advantages are reported for PDT, like inhibition of drug resistance pathways, low systemic toxicity, and ease of combination with other therapeutic regimens. All these advantages make PDT a more promising alternative to conventional treatment methods like chemotherapy, radiotherapy, and surgery [16,17]. Like every therapeutic modality, photodynamic therapy also has some limitations. One of PDT’s major limitations is the inability to be applied to the whole body in case of metastasis. So, its utilization is limited to precancerous lesion treatment and regional malignancies. In addition, treatment with PDT mainly relies on the presence of molecular oxygen in cancerous cells, and the singlet oxygen generated during PDT has a short half-life. Another limitation is its ineffectiveness in large tumor masses and deep cancers due to the limited light penetration [18]. Moreover, the PSs used in PDT have several drawbacks, such as skin photosensitivity, accumulation in healthy tissues, and limited selectivity for cancer cells [2,12,19]. Thus, photodynamic therapy has several advantages over conventional methods such as chemotherapy, radiotherapy, and surgery. The main advantages and disadvantages of PDT and other conventional therapies for cancer treatment are summarized in Table 1**.**

A solution to limit PDT’s disadvantages and make the most use of them is through designing nanoplatforms. Nanoplatforms have been designed to enhance existing photosensitizers to increase treatment efficiency by addressing the concerns of optical absorption, tumor targeting ability, and limited solubility [12,26]. Nanomaterials have promising optical properties that can improve the efficacy and enhance the penetration of PDT. Another use of nanomaterials is the encapsulation of photosensitizers to the nanocarriers, leading to high loading efficiencies, in addition to their surfaces, which can be decorated with versatile, functional groups to escort PSs selectively to the intended sites of action. These targeted PDT nanocarriers can have a role in reducing their side effects and improving their curative effects. Moreover, nanoparticles are stable under irradiation, unlike conventional organic PSs, which may exhibit a reduction in rates due to their instability upon irradiation exposure [6].

Interestingly, some nanomaterials, such as semiconductor nanoparticles, can serve as PS carriers and, at the same time, act as reactive oxygen species (ROS) producers [6,26]. Among the various NPs, silica nanoparticles have been given enormous consideration due to their unique features, such as a uniform pore size and large surface area, the presence of external and internal pores, the ease of surface modification, high mechanical and thermal stability, stable aqueous dispersion, high biocompatibility and biodegradability, and a high loading capacity [27,28]. Herein, this review will highlight and discuss the basic principles and current challenges of using photodynamic therapy in cancer treatment and how their therapeutic significance can be improved by using silicon-based inorganic nanoparticles.

## 2. Photodynamic Therapy (PDT) and Photosensitizers (PSs)

**Figure 2:**
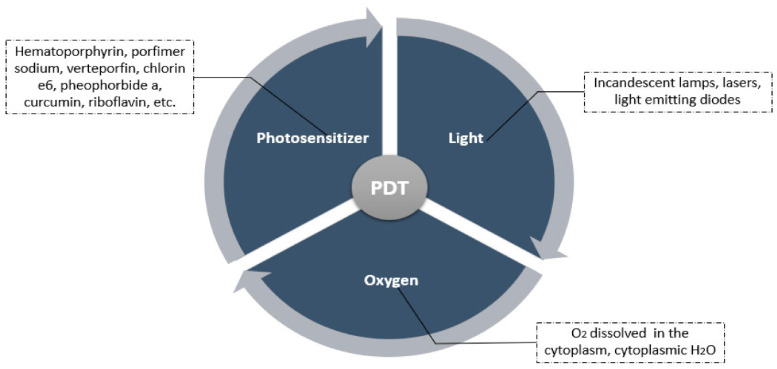
A summary of the main components of photodynamic therapy [12].

Because of its efficacy and cost-effectiveness, photodynamic therapy seems to be an attractive option for cancer treatment [28]. It has been investigated for more than 25 years as an untraditional treatment process for cancer [3]. This technique integrates three components: a photosensitizer, a light source with an appropriate wavelength, and molecular oxygen inside the tissues [28,29]. These three main elements are summarized in Figure 2.

**Figure 3:**
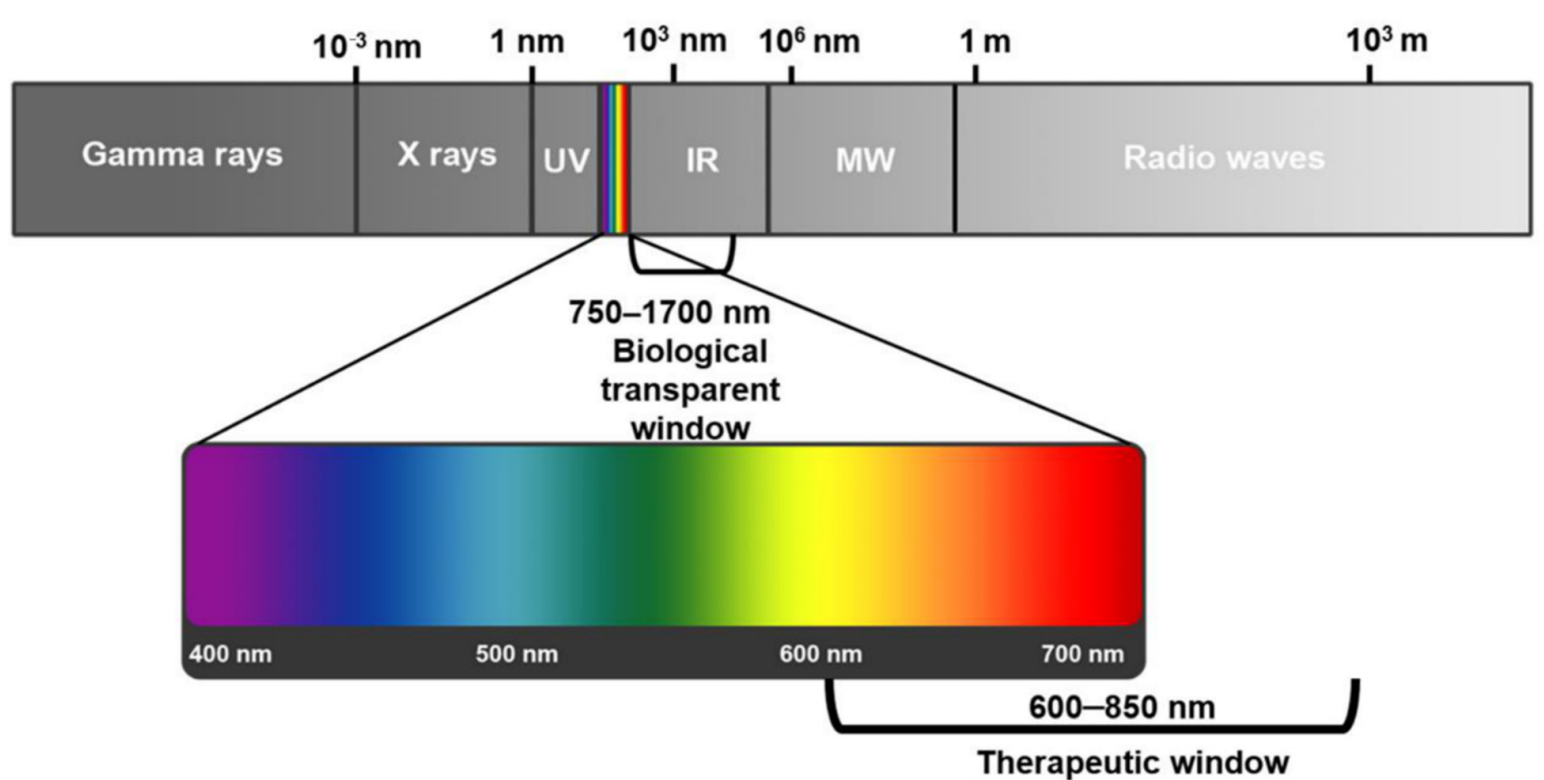
Electromagnetic spectrum showing the phototherapeutic window for photodynamic therapy for cancer treatment [30].

The light penetration into tumor cells is sophisticated; it can be scattered, reflected, or absorbed. The extent of these processes depends mainly on the light’s wavelength and the tissue type [13]. First, the endogenous chromophores in tissues such as myoglobin, cytochromes, and chromophores, which are responsible mainly for light absorption, can compete with photosensitizers and reduce the PDT process’s effect and reaction rate. Second, the tissue light absorption is inversely proportional to the wavelength of the light. So, the optimum reported wavelength to be used against cells ranges between 600 and 1200 nm, often called the tissue optical window [13,30]. Shorter wavelengths (<600 nm) do not have enough energy to produce enough reactive oxygen species and have a lower penetration depth in tissues, while longer wavelengths (>850 nm) are not sufficient to activate PSs [30]. Therefore, the most appropriate wavelength for PDT is between 600 and 850 nm, which is called the “phototherapeutic window” (Figure 3) [12,28].

The second key component in PDT is molecular oxygen. The oxygen concentration in the tumor tissues mainly affects the PDT treatment’s effectiveness. The oxygen concentration varies between different types of tumors and even between different regions of the same tumor [13]. Oxygen is decisive for the production of ROS during PDT treatment. However, hypoxia is the main pathological feature of many types of solid tumors, which leads to an imbalance between oxygen delivery and oxygen consumption. Hypoxia affects the success of cancer treatment via PDT due to the absence of molecular oxygen, resulting in poor treatment outcomes [31,32].

Another essential component of photodynamic therapy, apart from oxygen and light, is the type of PS. A PS works by absorbing electromagnetic radiation’s visible or ultraviolet region and then transferring this radiation to the adjacent molecules with a specific efficacy [8]. An ideal PS should be water-soluble [33], biocompatible, stable in plasma or serum solution [12], easily eliminated from the human body, and inactive in the absence of light radiation [12,13,33]. It must also possess a low photobleaching quantum yield, high intersystem crossing efficiencies [26], and finally, the ability to absorb light in the long-wave part of the spectrum (600–850) nm [1,30].

There are a variety of molecular structures of photosensitizers that are currently used in photodynamic therapy. They are divided into three generations of photosensitizers [13].

The first generation of PSs comprises various forms of hematoporphyrin derivatives (HPDs), used for treating cancer in the 1950s [18]. Photofrin (parfimer sodium), a hematporph derivative, has been involved in treating several types of cancer, such as cervical, bladder, esophageal, lung, and brain cancers [34]. However, despite their wide applications, first-generation photosensitizers have several disadvantages, including skin photosensitivity [12,33], ineffectiveness at low doses [6], instability, and nonselectivity. Another limitation of this generation is that they are excited only by short wavelengths, leading to low tissue penetration [34].

The second generation of PSs was developed in the late 1980s to overcome the first generation’s drawbacks [12]. Typically, the second-generation PSs are macrocyclic compounds derived from porphyrin moieties such as chlorins, phthalocyanines, bacteriochlorins, benzoporphyrin, temoporfin (Foscan), talaporfin, 5-amino levulinic acid, methyl aminolevulinate, and verteporfin [35,36,37]. In contrast to first-generation photosensitizers, these porphyrinoid compounds have faster elimination rates from the body. In addition to being more benign, they can deeply penetrate cancer tissues and absorb a spectrum ranging between 650 and 800 nm. However, they still cause tissue damage because of their higher toxicity and hydrophobic nature, which dictates the development of the third generation of PSs [12,18].

The development of third-generation photosensitizers mainly focuses on synthesizing structures with a higher affinity to cancer cells to facilitate cell uptake and improve the outcome of the therapeutic process [12]. The third generation mainly depends on modifying some available drugs with a specific agent, such as antibodies, amino acids, carbohydrates, etc., as targeting moieties. Another option is encapsulating second-generation photosensitizers in biodegradable or biocompatible nanocarriers. These modifications increase their accumulation at the target tissues [17,38].

Choosing an optimal photosensitizer is very important for the success of any photodynamic therapy. Despite many studies being performed using different types of photosensitizers, only a few PSs have received the U.S. Food and Drug Administration (FDA) approval for clinical use [39].

The selected PSs approved by the FDA are listed in Table 2.

## 3. Photodynamic Therapy (PDT) Mechanisms

**Figure 4:**
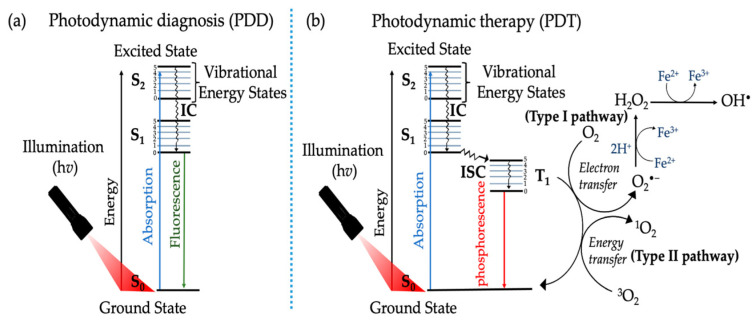
Modified Jablonski energy diagram, representing the photochemical and photophysical reactions of photosensitizers in (**a**) photodynamic diagnosis (PDD) and (**b**) photodynamic therapy (PDT) [5].

Photodynamic therapy combines photochemical and photophysical processes, ultimately improving therapeutic effects. Figure 4 illustrates the principle of PDT using the modified Jablonski energy diagram. Upon light absorption, the photosensitizer in the ground state (S_0_) is initially transformed into an excited singlet state (S_1_), leading to two possible processes. In the first possibility, the PS in the excited singlet state S_1_ is supposed to have a very short lifetime, so it returns to the ground state by emitting photon energy in the form of fluorescence emission. This type of photophysical process is used for photodynamic diagnosis (PDD) (Figure 4a) [5,6]. The second possibility is when the multiplication of the PS is transferred from the singlet state to the triplet state (T_1_) through intersystem crossing (ISC) to reach the first triplet excited state (T_1_). More importantly, the triplet excited state has a longer life when compared to the singlet excited state (S_1_) of a photosensitizer (Figure 4b) [6,35].

Photodynamic therapy can be classified into two major photochemical pathways (Type I and Type II). Both pathways lead to ROS and free radical generation upon activation with PDT process [5,41]. Electron transfer or/and hydrogen abstraction occurs between the substrates and photosensitizers for type I, generating free radicals. These formed radicals have a very short lifetime. They simultaneously react with molecules such as oxygen and water, producing hydrogen peroxide (H_2_O_2_), hydroxyl radical (·OH), and superoxide anion (O^2−^). As for the Type II process, the excitation energy is transferred to molecular oxygen (O_2_), forming singlet oxygen (^1^O_2_). The singlet oxygen is highly reactive and can interact with many biological substrates, eventually leading to cell death [13]. The photosensitizers can use either Type I, Type II, or both types of reaction instantaneously to destroy cancerous cells. The ratio between these two processes depends mainly on the oxygen concentration, substrates, photosensitizer, and the binding affinity of the photosensitizer to the substrate [6,13]. The therapeutic efficacy of type II PDT primarily depends on the tissue oxygen level and can only be initiated under well-oxygenated conditions. However, the hypoxic environment is a native microenvironment in solid tumors due to the rapid tumor growth and insufficient oxygen supply [11].

### The Effect of Photodynamic Therapy on Tumors

**Figure 5:**
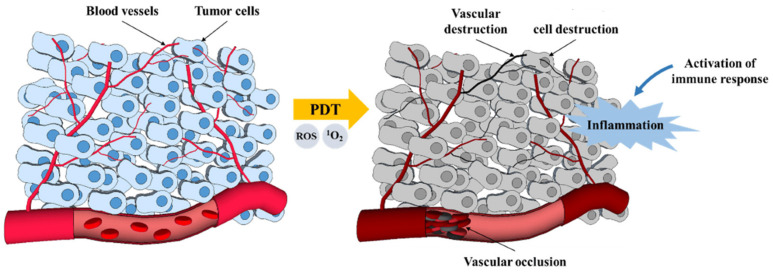
PDT mechanisms for tumor destruction [13].

The destruction of tumors by photodynamic therapy is a phenomenon that has been known for about a century. There are three mechanisms by which PDT mediates tumor destruction (apoptosis and necrosis, vascular mechanism, and immunological mechanism) (Figure 5). PDT stimulates the immune system against tumor cells, while conventional treatment methods such as chemotherapy and radiotherapy mainly cause immune suppression. PDT can induce inflammation, facilitate the antitumor T lymphocytes activation, and recruit leukocytes to target areas. It is also capable of killing tumor cells directly via necrosis induction (nonprogrammed cell death) or apoptosis (programmed cell death) by (^1^O_2_). Another reported effect of PDT is a reduction in the nature of the tumor microvasculature, from which the nutrients and oxygen in the tumor tissues are derived [13,36]. The three main mechanisms by which PDT mediates the destruction of tumor cells are summarized in Figure 5.

## 4. Nanotechnology in Photodynamic Therapy

Current research is trying to explore new strategies to improve photodynamic therapy (PDT) and lessen its limitations. One of the promising strategies is the integration of photosensitizers with nanotechnology. This approach has become a highly effective solution to increase therapy effectiveness. In recent years, various types of nanoparticles have been developed to be used as imaging probes or drug carriers due to the following advantages [12,42]: First, their large surface-to-volume ratio allows for the administration of a high amount of the drug, improves its delivery, and increases the uptake concentration in the target cells [7,30]. Second, the smaller size of nanoparticles makes them suitable for intravenous injection and also helps them mimic the biological molecules, enabling this nanosystem to pass easily through the immune system barriers. The third advantage is their surface, which can be functionalized with specific ligands. Surface functionalization using some moieties and functional groups enhances their ability to bind to a specific receptor on the target cells, increasing their specificity [12,42,43,44]. They can also prevent the antitumor drugs’ enzymatic inactivation or degradation by the plasma components like the overexpression of drug-metabolized enzymes (DMEs) [2,7]. PS integration with nanomaterials can offer a broader range of wavelengths, including ultraviolet, infrared, visible, and X-ray radiation. They can also increase the solubility of hydrophobic antitumor drugs by physical loading and chemical conjugation processes [26,45,46,47]. These advantages have made nanoparticles (NPs) a promising carrier system for the photosensitizers in PDT.

Depending on the nature of the PS, different strategies can be followed for their conjugation or attachment to the nanocarriers. Some examples of these strategies are utilizing physical adsorption, covalent conjugation, hydrophobic interactions, and encapsulation. The advantages and disadvantages of loading a photosensitizer into the organic and inorganic nanocarrier mainly depend on the characteristics or properties of the nanoparticles themselves [26,48].

On the one hand, organic nanostructures such as micelles, liposomes, dendrimers, polymeric [49,50], and lipid nanoparticles are especially favorable for encapsulating hydrophobic drugs. They can also be used in drug delivery applications due to their remarkable biopharmaceutical properties, such as good bioavailability, low systemic toxicity, controlled drug release, good water solubility, and degradation resistance [51]. On the other hand, inorganic nanosystems such as metal oxides (iron oxide, titanium dioxide, zinc oxide, etc.), mesoporous silica, semiconductors (quantum dots, graphene), and metallic nanoparticles have also been used successfully for drug delivery. Inorganic nanosystems are distinguished by their chemical maintenance, thermal stability, good bioavailability, possibility to improve the therapeutic action of loaded drugs, and low toxicity [52].

### 4.1. Silica Nanoparticles

The encapsulation of PSs using silica nanoparticles is one of the most attractive strategies in PDT [12]. Silica is one of the most abundant elements in the earth’s crust and is produced from various sources [53]. It is an oxide of silicon and is considered one of the most efficient carriers for drug delivery control [30]. In general terms, silica nanoparticles are well known for their usually higher colloidal stability, biocompatibility, and ability to produce ^1^O_2_, as well as their functionalization properties [26,54]. Silica nanoparticles can be classified into three different types: (1) Stöber silica nanoparticles (nonporous) without porous structure, (2) organically modified silica (Oromosil), an organophilic core produced by the combination of different silica sources, and (3) mesoporous silica nanoparticles (MSNs) with a porous structure (pore size: 2–50 nm). These different types of silica nanoparticles are differentiated based on their structures or their core nature [26]. Among these three types, mesoporous silica nanoparticles (MSNs) are considered suitable candidates in photodynamic therapy. MSNs possess several interesting properties that make them one of the promising nanocarriers for the effective delivery of PSs in PDT. These features include the (i) ease of functionalization with targeting ligands, (ii) outstanding biocompatibility, (iii) large surface areas (>1000 m^2^/g), (iv) high loading efficiencies of different therapeutic agents, (v) tunable particle sizes and morphologies [55,56], and (iv) optical transparency, which are crucial for PDT [27,57,58,59,60]. In addition, MSNs have been reported to address some of the challenges accompanying the use of PSs in PDT, such as poor selectivity to tumor tissues, hydrophobicity, and long-term accumulation in healthy tissues. In the coming sections, we will review the most recent studies that utilized MSNs as a promising nanocarrier for PSs, aiming at improving PDT.

### 4.2. MSNs in Photodynamic Therapy

**Figure 6:**
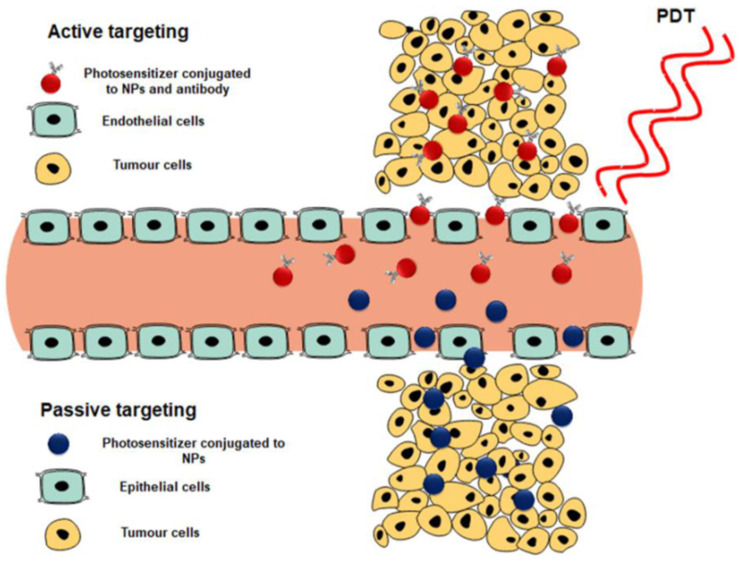
Active and passive targeting of photosensitizers to generate single oxygen and reactive oxygen species for tumor destruction [30].

Photosensitizer subcellular localization uptake can be classified into either active or passive targeting processes (Figure 6).

Active targeting is achieved by decorating the surface of the photosensitizer-loaded nanocarrier system with a specific targeting ligand. This functional decoration can be via peptides, antibodies, folic acid, aptamers, carbohydrates, or small ligands, which are overexpressed only on cancer cells. As a result, the PS uptake in these cells is actively internalized and specifically enhanced [59]. On the other hand, passive targeting can occur via the permeability and retention effect (EPR), causing a leaky vasculature on tumor tissues [59,61]. 

This naturally occurring process utilizes the difference in pathophysiological and anatomical abnormalities between cancer tissue and normal cells. The active targeting of nanoparticles provides a more selective absorption of photosensitizers with an increased concentration accumulation [30].

### 4.3. Photosensitizers Loaded on MSNs

Two main groups of silica nanocarriers can be primarily found in this review. On the one hand, silica nanoparticles are often used as a coating for other nanostructures. The second type is silica-based nanomaterials that are involved in the photosensitizer’s incorporation, making this type the fourth generation of photosensitizers [26]. In this review, we have focused on recent work on the applications of silica nanoparticles as photosensitizer vehicles for photodynamic therapy.

The following sections show a more profound view of the most representative examples of loading PSs onto silica nanoparticles. These sections are divided according to the type of photosensitizer used.

#### 4.3.1. Porphyrin Photosensitizer

Porphyrins are macrocyclic pigments, commonly occurring in nature. They are often known as pigments of life. Porphyrin’s name comes from porphyra, a Greek word that means purple, because porphyrins are usually red or bright purple. Porphyrins are widely used in PDT owing to the unique photosensitive porphyrins [62]. In fact, several studies were carried out with porphyrins as photosensitizers in PDT. First, Elisa Bouffard et al. developed highly efficient targeting molecules of mesoporous silica nanoparticles (MSNs), carrying porphyrin as the photosensitizer for photodynamic therapy targeting prostate cancer. The surface of NPs is decorated with diamino-side carboxylate, which can specifically bind to mannose-6-phosphate receptors as it is overexpressed in prostate cancer. This targeted PS via porphyrin leads to a higher endocytosis of tumor cells [63]. Dina Aggad et al. reported ethylene-based periodic mesoporous organosilica nanoparticles (PMOs) for PDT and the autonomous delivery of gemcitabine hydrochloride, an FDA-approved chemotherapeutic drug, in cancer cells. Depending on the nature of the photosensitizer (tetrasiylated porphyrins or monosilylated porphyrin) and its aggregation state, they could form two-photon PDT. The synergistic effect of two-photon irradiation with gemcitabine raised the percentage of cell death by about 20% compared to the delivery process without irradiation [64]. Si Li et al. were also able to design mesoporous silica nanoparticles (MSNs) as a nanocarrier composed of silica nanoparticles (SiNPs), 5,10,15,20-tetrakis (1-methyl 4-pyridinio), and porphyrin tetra (p-toluenesulfonate) (TMPyP) photosensitizer, and this was further decorated with folic acid (FA). Under light irradiation near-infrared light (NIR = 655 nm), the embedded TMPyP could generate singlet oxygen. Additionally, the doxorubicin (DOX) anticancer drug could be loaded onto them for chemotherapeutic purposes. In vitro cytotoxicity assay shows that the viability of MCF-7 breast cancer cells, the most common type of cancer among women, treated with MSN@SiNP@TMPyP-FA/DOX under NIR irradiation was lowered by 30% [65]. In a different study, the periodic mesoporous organosilica nanoparticles (PMOs) encapsulated with protoporphyrin IX (PpIX) photosensitizer molecules showed a significant PDT effect in colon carcinoma (HT-29) and *Esherichia coli* (*E. coli*), a Gram-negative bacterial strain. These nanoparticle frameworks were beneficial for in vitro PDT on human colon cancer cells [66]. Jiefei Wang et al. managed to develop new nanoparticles by assembling porphyrin as the core surrounded by convenient functionalization. The designed nanoparticles improved their biocompatibility with the cancer cells and their ability to generate lethal singlet oxygen ^1^O_2_ [67]. Also, silica was reported to be used in coating gold nanorods (AuNRs), conjugated with 4-carboxyphenyl porphyrin (TCPP), to form AuNR@SiO_2_-TCCP where AuNRs act as a photothermal agent, while TCPP acts as a photosensitizer. Like the ones developed by Wang et al. [67], this prepared nanoparticles possess good biocompatibility and can generate single oxygen efficiently. The results illustrated that the AuNR@SiO_2_-TCPP showed a high synergistic effect in photodynamic and photothermal therapy against cancer cells in both in vivo and in vitro studies [68]. Rod-like nanomaterials were also studied in different studies where rod-like mesoporous silica nanoparticles coated with gold nanoshell and modified with ultrasmall gadolinium (Gd)-chelated 5,10,15,20 tetrakis (4-sulfonat-o-phenyl) porphyrin (TPPS4) photosensitizers were designed. This designed nanostructure could control PTT/PDT combined anticancer therapy. Also, nanosheets were used, where gold nanosheet was found to act as a perfect PTT agent, and a TPPS4(Gd) photosensitizer was able to generate singlet oxygen ^1^O_2_ with high efficiency [69].

#### 4.3.2. Phthalocyanines Photosensitizers

Phthalocyanines are a class of promising second-generation photosensitizers that exhibit higher singlet oxygen quantum yields and have strong absorption in the near-infrared region (NIR) [60].

A study conducted by Ozge Er et al. described the loading of zinc phthalocyanine (ZnPc) onto mesoporous silica nanoparticles (MSNPs) for in vivo and intracellular PDT against pancreatic cancer cells. The histopathology studies revealed that the necrosis of tumor cells was higher in the treated group than in the control group. The surface modification of MSNPs using polyethylene glycol (PEG) led to an increase in the blood circulation time, a decrease in the extraction rate of these nanophotosensitizers, and a decrease in the labeling process [70]. In another study, a functionalized zinc (II) phthalocyanine (ZnPc) was conjugated with stellate mesoporous silica nanoparticles (SMSNs) through the formation of an acid-sensitive hydrazone bond. This nanosystem (SMSN-ZnPc) was activated inside tumor cells, and ROS were generated efficiently against Hela cells. It can also exhibit efficient tumor growth inhibition with negligible systemic toxicity [71]. Özge Er et al. also developed mesoporous silica nanoparticles loaded with zinc (II) 2,3,9,10,16,17,23,24-octa (tert-butyl phenoxy phthalocyaninato (2-)-N29,N30,N31,N32 (ZnPcOBP) as a photosensitizer to determine the production of singlet oxygen and achieve in vitro PDT against pancreatic cancer cells. When ZnPcOBP was incorporated in silica nanoparticles, it showed a high phototoxic effect, which was enhanced by cetuximab. Cetuximab is a monoclonal antibody that mainly targets the epidermal growth factor receptor (EGFR). So, the imidazole was an excellent vehicle for the selective delivery of ZnPcOBP to pancreatic cancer cells (ASPC-1, PANC-1, MATpaca-2) in vitro [72]. Yiming Zhou et al. reported ultrasmall PH-responsive silicon phthalocyanine nanomicelles (PSN0), which were designed for selective PDT against tumors, with minimum damage to normal tissues. The results of in vivo studies declared that PSN, as a pH-responsive photosensitizer, has broad applications depending on the selectivity of the PDT against tumor cells. It also reported that the tumor cells were eliminated without recurrence [73]. A study conducted by Tingting Shen et al. aimed to improve ROS production in specific H1299 tumor locations via loading gold nanoparticles (AuNPs) onto the surface of mesoporous silica-coated upconversion nanoparticles. This nanosystem was loaded into the silica shell with a photosensitizer (silica phthalocyanine dihydroxide) and small molecules DC50 (C17H14BrF2N3OS). This nanoplatform exhibited a specific cytotoxic effect by expressing Atox1 and CCS proteins after internalization by specific tumor cells [74]. Burcu GÜleryÜz et al. developed a (MC540/ZnPc-UCNP@Au) nanoplatform through the synthesis of upconversion nanoparticles (UCNP) to convert near-infrared light into multiple visible wavelengths, coated with porous silica nanoparticles and then uploaded with a dual photosensitizer merocyanine 540 (MC540), zinc phthalocyanine (ZnPc), and gold (Au) functionalization to enhance PDT treatment against prostate cancer cell (PC3). MC540/ZnPc-UCNP@Au nanoplatforms could transform near-infrared (980 nm) to visible (540 and 660 nm) to activate PSs. In addition, this nanoplatform allows for treatment of a deep-seated prostate cancer cell due to the high penetration property of NIR light [75].

Another commonly used second-generation PS is phthalocyanine (Pc), which was re-formulated, forming benzyl ester dendrimer silicon phthalocyanine (D-SiPc). In order to improve the cell permeability of D-SiPc, it was loaded on amphiphilic block copolymer (methoxy polyethylene glycol-polylactic acid, MPEG_500_-PLA_300_) to form polymeric nanoparticles. These polymeric nanoparticles exhibited a high photo-cytotoxic effect against U251 glioma cells under laser irradiation [76]. Xiuqin Chen et al. developed a cholesterol silicon (IV) phthalocyanine (Chol-Pc) photosensitizer based on DSPE@Chol-Pc nanoparticles, using DSPE-PEG_500_ as a nanocarrier. This nanosystem could effectively produce reactive oxygen species (ROS) and exhibited a phototoxic effect on (MCF-7) breast cancer cells, leading to cell death by destroying the cholesterol-rich membrane. DSPE@Chol-Pc is mainly distributed in rich cholesterol cells, especially in the Golgi apparatus of breast cancer cells [77].

#### 4.3.3. Chlorin e6 Photosensitizer

Chlorin e6 (Ce6) is an FDA-approved second-generation photosensitizer that meets the desired clinical properties for photodynamic therapy. It is characterized by a high reactive oxygen species generation ability and high anticancer potency against many types of cancer cells. The major drawback of Ce6 is its hydrophobicity, leading to its rapid clearance from the circulatory system and poor biodistribution. Several nanosystems have been designed to overcome this drawback and enhance Ce6 bioavailability [78,79]. Xuemel Wang et al. reported redox nanocarriers (RN), which are prepared using hollow mesoporous silica nanospheres decorated with a redox polymer ligand. This designed structure is characterized by high biocompatibility and low in vitro cytotoxicity. This nanocarrier was loaded with catalase and metformin, which is responsible for inhibiting the mitochondrial respiration of cancer cells, reducing the activity of tumor cells, and increasing the concentration of oxygen in PDT, upon its linkage with chlorine e6 photosensitizer [80]. In another study, MSNs have been investigated for controlling drug delivery to the target site. This system consists of two main constituents: a gadolinium complex (Gd-DOTA) that acts as the ROS gatekeeper and polyethylene glycol (PEG) conjugated with Ce6, which acts as the ROS generator. Both constituents are important for magnetic resonance imaging to guide photodynamic chemotherapy. It also contains doxorubicin (Dox), a chosen anticancer drug that is responsible for maintaining the structural integrity and enhancing the in vitro imaging signal. DOX-R-MSNs, under 660 nm laser irradiation, exhibit a rapid DOX release at the tumor site, effectively inhibiting tumor growth [81]. Xiaoli Cai et al. developed promising three-dimensional dendritic mesoporous silica nanoparticles to deliver a novel hydrophobic photosensitizer (Chlorin e6) to lung cancer cells (A549). The nanoenzymes (platinum (Pt) nanoparticles) were immobilized into the channels of the nanosystem to catalyze the conversion process of hydrogen peroxide (H_2_O_2_) to oxygen intracellularly. Also, the surface was decorated with triphenylphosphine (TPP) to target the mitochondria. MTT assays showed that more than 80% of the cancer cells died after treatment with Pt-DMSN-TPP/Ce6 nanoparticles irradiated with 660 nm [82]. Qi Sun et al. first reported that gold (Au) nanorods were capped to chlorine e6-doped mesoporous silica nanorods for the single wavelength of NIR light-triggered combined phototherapy (photodynamic therapy (PDT) and photothermal therapy (PTT)). This single wavelength of light and the rod shape of the nanosystem for the combined phototherapy had the following remarkable features: (1) the effect of PDT and PTT could be achieved under a single wavelength of near-infrared light (660 nm), which makes the therapeutical process feasible and simple and (2) chlorin e6 doped into a mesoporous nanorod without being released from the nanocarrier during delivery. AuNRs-Ce6-MSNRs are not only able to produce single oxygen for PDT based on chlorin e6 after the uncapping of gold nanorods under the single NIR irradiation, but they are also able to generate heat to perform the PTT effect based on AuNRs [83]. In another study, the photosensitizer chlorin e6 was covalently attached to the external and internal surfaces of MSNs. Then, the nanoparticles were anchored by triphenyl phosphonium for selective mitochondrial targeting. When the nanophotosensitizer was irradiated with a laser (655 nm, 0.1 W.cm^−2^), it generated a large amount of ROS in the mitochondria, leading to mitochondrial dysfunction and cell apoptosis [84]. Zhen Lu Yong et al. reported a multifunctional and safe oxygen-evolving nanoplatform composed of Prussian blue core and chlorin e6-anchored periodic mesoporous organosilica shell (PB@PMO-Ce6). The Prussian blue (PB) can catalyze hydrogen peroxide (H_2_O_2_) to generate molecular oxygen (O_2_), and the chlorin e6, upon laser irradiation, becomes able to transform the produced molecular oxygen O_2_ to a more reactive oxygen species (ROS). The histopathological analysis showed that this nanoplatform could elevate singlet oxygen to effectively inhibit the growth of tumor cells without obvious damage to major organs [85]. Sanghyo Park et al. developed MSNs that are conjugated with hyaluronic acid (HA) for specific cancer cell targeting. Also, the prepared HA-MSNs exhibited a high drug-loading capacity and sustained drug release. To enhance the anticancer effect of PDT, chlorin e6 (Ce6) and doxorubicin (DOX) were loaded in HA-MSNs to form a (DOX/Ce6/HA-MSNs) nanoplatform, which exhibited highly effective cytotoxicity on squamous cell carcinoma 7 (SCC7) compared to the corresponding free drug [86]. A smart multifunctional nanoplatform was designed by coating core–shell composite mesoporous silica-encapsulated upconversion nanoparticles and chlorin e6 (Ce6) with degradable calcium phosphate, and then this system was loaded with doxorubicin (DOX). This nanoplatform has the synergistic effect of ROS, which mediates drug release to achieve a high therapeutic effect. Based on this experiment’s results, these nanoplatforms conjugated with chlorin e6 photosensitizer can significantly enhance the cellular uptake and the photodynamic therapy response in both in vivo and in vitro processes. Moreover, it improved the selectivity of nanosystems towards cancer cells [87]. Additionally, hollow mesoporous silica nanospheres were loaded with doxorubicin (DOX) and chlorine e6 (Ce6). Afterwards, bovine serum albumin (BSA) and integrated manganese dioxide (MnO_2_) formed a BA-MnO_2_ nanoparticle that was anchored to the surface of a loaded hollow mesoporous silica nanoparticle through the formation of disulfide bonds to form (BSA-MnO_2_@HMSNs-DOXCe6, BMHDC). Such a nanoplatform exhibited a loading capacity effect (36% of Ce6 and 14% of DOX) and pH sensitivity via Ce6 and DOX behavior through the breakdown of disulfide bonds [88]. A different study successfully developed hollow MSNs co-loaded with manganese oxide NPs and chlorine e6 for tumor magnetic resonance imaging (MRT) and in vivo PDT. This universal “on/off” switching strategy was found to be able to control the loading amount of Ce6 and Mn. The results have shown that the increase in O_2_ concentration improved PDT efficiency and exhibited effective tumor inhibition, reducing the side effects on normal cells [89]. Jiaxing Yang et al. developed silica nanoparticles decorated with cell membrane (CM) derived from SGC7901 cells to specifically target gastric cancer (homogenous SGC7901) in vivo and in vitro. The designed CM/SLN/Ce6 diameter was about 115.6 nm, with a surface charge of −30.4 mv [90]. Another recent study reported successfully fabricated upconversion nanoparticles (UCNPs) with a dual nature (ROS and pH) to utilize chlorin e6 (Ce6) and doxorubicin (DOX). The DOX and Ce6 were conjugated in a 1:1 (w:w) ratio and then loaded onto the surface of UCNPs@mesoporous silica nanoparticles with a diameter of 85.63 ± 9.87 nm. Ce6 controlled the release of DOX under NIR laser irradiation at 980 nm. A cytotoxic study showed that this modified nanosystem could successfully deliver DOX and Ce6 at a specific tumor site, causing cell death [91]. Xiang Long Tang et al. developed a Fe_3_O_4_@mSiO_2_(DOX)@HSA(Ce6) nanoplatform where doxorubicin (DOX) molecules were loaded onto Fe_3_O_4_/mSiO_2_ to form Fe_3_O_4_ mSiO_2_(DOX), and then polydopamine (PDA) was coated onto Fe_3_O_4_/mSiO_2_(DOX) to obtain PDA-coated Fe_3_O_4_/mSiO_2_(DOX). Human serum albumin (HAS) was conjugated to the other surface of the nanosystem to increase the blood circulation time and biocompatibility and act as a vehicle for the chlorin e6 photosensitizer [92]. Hu et al. developed upconversion nanoparticles (UCNPs) that encapsulated chlorin e6 (Ce6) and the glyican-3 antibody (GPC3), which is overexpressed in hepatocellular carcinoma cells. This nanoplatform had great anticancer potential and was strongly expected to be used in liver cancer treatment [93].

#### 4.3.4. Indocyanine Green Photosensitizers

Indocyanine green (ICG) is a near-infrared tricarbocyanine dye approved by the FDA for human clinical use. Indocyanine green is a photosensitizer that kills tumor cells by producing photothermal heat or singlet oxygen. Its limitations include rapid aqueous degradation, a short half-life time, and high aggregation rates. To address these limitations, ICG is formulated with NPs [94]. For instance, Yuting Hung et al. developed dendritic mesoporous organosilica nanoparticles (MONs), which can be used to encapsulate indocyanine green (ICG) photosensitizer on macromolecular catalase (CAT) to overcome hypoxia of tumor cells upon 808 nm laser irradiations. ICG can generate highly cytotoxic (^1^O_2_) singlet oxygen and ROS to release photoacoustic imaging and kill cancer cells [95]. In a different study, the ICG was incorporated with doxorubicin hydrochloride into hollow mesoporous silica nanoparticles and dopamine-modified hyaluronic acid (DA-HA) to act as targeting agents and gatekeepers linked to HMSNs via boronate ester bonds. In vitro cell culture experiments showed that the ID@HMSNs-B-HA nanoplatform (where ID represents both DOX and ICG) could inhibit murine mammary carcinoma cells (4T1) via a combination of PDT with chemotherapy [96]. Chenlu Huang et al. also reported mesoporous silica nanoparticles (MSNs) encapsulated with perfluorohexane (PFH) with indocyanine green as photosensitizer, coated with a polydopamine (PDA) layer and polyethylene glycol-folic acid. MSNs-PFH@PDA-ICG-PEG-FA had a good monodispersity, with enhanced ICG photostability and cellular uptake. Upon irradiation with 808 nm NIR, the nanocarrier produces ROS for effective PDT and generates hyperthermia to realize PTT [97]. Cui-E-Shi et al. designed mitochondria-targeted hollow mesoporous silica nanoparticles (THMSNs) loaded with phase change material L-menthol (LM). Meanwhile, an ICG photosensitizer and doxorubicin (DOX) were encapsulated into THMSNs. The formed THMSNs@LMDI has improved the specific accumulation in mitochondria and cellular internalization [98]. Another study reported mesoporous silica nanoparticles encapsulation with indocyanine green and α-tocopherol succinate to reduce innate oxygen consumption by blocking the mitochondrial respiration chain. The MSN was modified with phenyl phosphine, enhancing blood circulation and mitochondrial target specificity. This nanosystem offers a promising nanoplatform to overcome hypoxia, which is responsible for limiting PDT application [99]. A study conducted by Bakai Zhang et al. reported gold nanorods (AuNRs) coated with SiO_2_ as a first dense layer, then with mesoporous SiO_2_ as a second mesoporous layer, forming Au@SiO_2_@mSiO_2_ to combine photodynamic therapy and photothermal therapy for multifunction theragnostic use. This nanosystem was loaded with indocyanine green (ICG), serving as an excellent agent for PDT and PTT. By using Au@SiO_2_@mSiO_2_-ICG, the ability to kill cancer cells increases three times compared to free ICG [100].

#### 4.3.5. Other Photosensitizers

Triple-negative breast cancer (TNBC) is considered an aggressive subset of breast cancer, and currently, no effective therapeutic drug has been identified. Tao Zhang et al. developed a biomimetic nanoplatform based on a leucocyte/platelet hybrid membrane (LPHM) and large-pore dendritic mesoporous silicon nanoparticles (DLMSNs). This system was loaded with doxorubicin (DOX) and fluorescent dye (IR = 780 nm) photosensitizer to prepare an LPHM@DLMSN@DOX/IR 780 nanoplatform, which effectively suppressed tumor growth [101]. In a different study, Triapazamine (TPZ) was used as a photosensitizer encapsulated in mesoporous silica nanoparticles (MSNs). MSNs were decorated with polyethylene glycol and folic acid to specifically target the activated macrophage and inhibit the progression of arthritis [102]. Another recent study reported on verteporfin (Ver), a second-generation photosensitizer conjugated with mesoporous silica nanoparticles. The resulting Ver-MSNs are considered an efficient nanoplatform, that can reduce or inhibit melanoma growth (skin cancer). In vitro experiments used melanoma cells irradiated with red light (693 nm) to decrease cancer cell proliferation [103]. The advanced stage of melanoma cancer is responsible for most cancer deaths. However, the survival ratio could be more than 90% if treated in the early stage [104]. Ka Chen et al. designed a multifunctional drug delivery system (RB-DOX@HMSNs-N=C-HA) to realize PDT. Hollow mesoporous silica nanoparticles (HMSNs) were used as a host material to encapsulate rose Bengal (RB) photosensitizer and doxorubicin. The surface of HMSNs was modified with hyaluronic acid (HA). The drug loading capacity was 12.78% for RB and 15.3% for DOX. The in vitro cellular uptake and cytotoxicity assay proved that the RB-DOX@HMSNs-N=C-HA nanosystem could efficiently target murine mammary carcinoma cells and inhibit tumor cell viability with combined chemo-photodynamic synergistic therapy [105]. Gaizhen Kuong et al. reported a mesoporous silica-based drug delivery system decorated with polyethylene glycol and curcumin (Cur) as photosensitizers to solve the low bioavailability of curcumin. The results showed that MSN-PEG@Cur generated ROS upon irradiation to get effective PDT in cancer treatment [106]. Another study reported the synthesis of MSNs to encapsulate the Ru (II) polypyridine complex, another type of photosensitizer. The surface of MSNs was decorated with folic acid, which acts as a targeting moiety for folate receptors, overexpressed in ovarian carcinoma cells. Upon irradiation of the nanophotosensitizer at 480 or 540 nm, the conjugate was nontoxic in normal tissue while showing a phototoxic effect in ovarian carcinoma cells [107]. A study conducted by Yanjun Yang et al. reported diselenide mesoporous silica nanoparticles modified with polyethylene glycol. These carriers encapsulated a methylene blue photosensitizer and doxorubicin chemotherapeutic drug. Under low doses of red-light irradiation during PDT, the ROS mediates a diselenide bond, resulting in the degradation of the organosilica matrix. Both drugs were released, resulting in a chemo-photodynamic therapy for breast cancer [108]. Zhibin Yin et al. developed mesoporous silica nanoparticles loaded with gold nanoclusters (AuNCs) and manganese dioxide (MnO_2)_ nanosheets, which were wrapped as switching shield shells (AuNCs@mSiO_2_@MnO_2_). Under irradiation with a 635 nm laser, the stable MnO_2_ shells eliminated the singlet oxygen generator _(_^1^O_2_) to switch off photodynamic therapy and magnetic resonance imaging. While in the acidic microenvironment of tumor cells, the MnO_2_ shell reacts with H_2_O_2,_ resulting in the degradation of MnO_2_ and generation of molecular oxygen O_2_ to enhance the PDT effect. Also, the cell viability of MDA-MA435 (metastatic human breast cancer) cells was reduced to 4%, and the tumor cells completely disappeared [109]. Dongxue Guo et al. developed a mesoporous silica nanoplatform, and its surface was decorated with gold nanoparticles. To improve the stability of the nanoplatform and control the drug release, the modified polyethylene glycol was introduced to the nanosystem to act as a gatekeeper. The formed MSN-Au-PEG nanoplatform loaded with DOX anticancer drug improved its effect in PDT [110]. Also, mesoporous silica nanoparticles were loaded with singlet oxygen (^1^O_2_) photosensitizer and DOX, and the surface of the nanoparticles was integrated with nitric oxide (NO) photodonor (NOPO). The complete nanoconstruct (PS-MSNs/NOPD/DOX) can deliver singlet oxygen and NO under green and blue light, respectively. Also, it helps release DOX under specific physiological conditions in A375 melanoma cancer cells [111]. Ting Sheng Yan et al. reported the formation of a biodegradable pH-sensitive hollow mesoporous silica nanoparticle (HMSN) for co-delivery of photosensitizer pheophorbide (PA) and an antitumor agent (DOX). This nanoplatform surface was decorated with folic acid to mediate endocytosis, which could effectively avoid the side effects on normal tissues [112]. The surface of the MSNs was decorated with iodine BODIPY containing a disulfide bond and a carboxyl group (COOH) for PDT. Also, PEGylation was performed to improve their water solubility by forming MSN-I_2_BOD-PEG. The formed nanoparticles could generate more reactive oxygen species to kill tumor cells and enhance endocytosis by Hela cells under 500 nm light irradiation [113]. In another study, Ruth Prieto Monter et al. also designed mesoporous silica nanoparticles loaded with BODIPY as a photosensitizer, and the surface was decorated with PEG and FA. BOD-PEG-FA under 518 nm irradiation (10 J/cm^−2^) could improve biocompatibility and generate more singlet oxygen to kill cancer cells [114]. Binbin Ding et al. developed monodispersed mesoporous silica-coated upconversion nanoparticles (UCNPs) to act as carriers to a specific photosensitizer MC540 and OVA (chicken oval albumin). Also, the nanoparticle’s surface was decorated with tumor cell fragments (TFs) as tumor antigens were developed. Under 980 nm NIR irradiation, this nanosystem possesses the best synergistic immunopotentiation action, verified by a high frequency of CD^4+^ and CD^8+^ and strong Th1 and Th2 immune responses [115]. Julia Elistratova et al. reported silica nanoparticles decorated with luminescent hexamolybdeum cluster complexes as photosensitizers. The prepared C-SNs hybrid nanosystem showed a significant PDT effect on the MCF-7 cancer cell line by a high potential ability to generate ROS [116]. Cichorium pumilum (CP) is a natural photosensitizer with many useful effects in treating cancer. However, its low bioavailability and poor water solubility have confined its use. These limitations can be addressed when it is encapsulated in silicon nanoparticles. The results showed that CP-SiNPs exhibited a high efficacy compared to free CP by a rise of 49.45% in the concentration effect [117]. Ya-Kun Dou et al. developed a ruthenium complex loaded onto silica nanoparticles, and the surface was decorated with folic acid. This synthesized SiNPs-Ru could emit red fluorescence by two-photon excitations, effectively killing cancer cells via PDT [118]. A recent study reported core–shell hybrid nanoparticles, formed by encapsulated xylan carrying a 5-(4-hydroxyphenyl)-10,15,20-triphenylporphyrin (TPPOH). In vitro analysis showed that the formed nanosystem is more effective against HCT116 cells and HT-29 (colorectal cancer cell lines) than free TPPOH [119]. Finally, Xufeng Zhu et al. developed a mesoporous ruthenium nanosystem with a dual targeting function. Aptamer AS441 (Apt) and transferrin (Tf) were grafted on the surface of a mesoporous ruthenium nanoplatform (MRN) with a higher loading capacity, and [Ru (bpy)_2_ (tip)] was used as a photosensitizer. Both targeting ligand and capping agents enable the effective penetration of the blood–brain barrier and target glioma cancer. Also, RBT produced reactive oxygen species and induced apoptosis in cancer cells when subjected to laser irradiation [120,121,122].

The application of silica nanoparticles as photosensitizer vehicles for photodynamic therapy is summarized in Table 3.

## 5. Conclusions and Future Prospects

This review described the most recent state-of-the-art studies reporting the use of integrated MSNs/PDT for improving cancer therapy. Photodynamic therapy is a breakthrough noninvasive therapy that relies mainly on the presence of molecular oxygen, light (with a specific wavelength), and PSs. Conventional PDT is limited by the depth of light penetration and the PSs’ limitations, such as nonselective targeting of the intended organs, hydrophobicity, cumulative toxicity, and poor cellular uptake. In this regard, several studies have reported the integration of nanocarriers to address the limitations of PSs and, hence, modernize PDT compared to the conventional one. MSNs set themselves apart from other types of organic and inorganic nanoparticles. This is attributed to their ease of fabrication, high loading capacities, ease of surface decoration with various functional groups, biocompatibility, and optical transparency. All the recent studies have proven that PSs combined with MSNs could show beneficial properties over free photosensitizers in a solution, such as by enhancing their selectivity and internalization in tumor cells. Additionally, silica nanoparticles can increase the solubility and stability of PSs in the physiological media and minimize or eliminate the cytotoxic effect in dark conditions. Recently, several studies have reported the application of MSNs in PDT for cancer treatment, which might revive hope for cancer patients.

Despite the endeavors in synthesizing biocompatible MSNs to serve as carriers for various PSs to improve cancer therapy, several challenges do exist that need to be addressed. Among these challenges is the shortage of enough in-depth knowledge on the in vivo biocompatibility and biodistribution of MSNs, their immunological reactions, biodegradation, bioelimination, and cumulative toxic effects. Future research should study the biosafety concerns related to the application of MSNs in cancer therapy rather than focusing only on their synthesis, functionalization, and optimization. For clinical applications, more in vivo mechanistic studies and clinical trials should be conducted to add a wealth of information to discover safe and effective cancer therapies. To date, and to the best of our knowledge, no clinical investigations involving the use of MSNs in combination with PDT in cancer therapy have been reported. The hallmark for the success of MSN-based therapies is including in vivo research and clinical trials that will be conducted in the coming years.

In conclusion, MSNs are prominent nanocarriers that supplement the conventional PDT. The hallmark for the success of MSN-based therapies is the inclusion of more in-depth in vivo studies and clinical trials in future studies. 

## Figures and Tables

**Table 1:** A summary of the advantages and disadvantages of photodynamic therapy and other conventional therapies in cancer treatment.

Therapeutic Option	Classification	Advantages	Disadvantages	Ref.
Photodynamic therapy	Localized	✓ Low side effects ✓ Noninvasive ✓ Short treatment time ✓ Cost-effective ✓ Can be targeted ✓ Can be repeated ✓ Cancer selectivity ✓ Excellent cosmetic outcome ✓ Immunogenic ✓ Nonpotential for resistance	Photosensitivity after treatment Tissue oxygenation is crucial for the photodynamic effect Treatment efficacy is dependent on the accuracy of tumor light irradiation It is challenging to treat metastatic cancers with current technology	[5,12]
Chemotherapy	Systemic	✓ Can reach malignant cells ✓ Light-independent ✓ Suitable for systemic cancers ✓ Many available drugs	Increases systemic toxicity Induction of multidrug resistance Hair loss and weight loss Induction of fertility complications Can lead to peripheral neuropathy or other nervous system complications	[20,21]
Radiotherapy	Localized	✓ Noninvasive when compared to surgery ✓ Low toxicity when compared to systemic therapies ✓ Cost-effective ✓ Convivence	Unsuitable for systemic cancers Increased need for imaging techniques Limited information on adverse effects High chances of inducing the development of secondary cancers	[22,23]
Surgery	Localized	✓ Quick and effective ✓ Light-independent ✓ Improves quality of life ✓ Cost-effective when compared to systemic therapy	Invasive and painful Wound bleeding and swelling Susceptibility to infections and metastatic tumors	[24,25]

**Table 2:** Selected FDA-approved photosensitizers for photodynamic therapy.

Chemical Name	Trade Name	Scaffold	Chemical Structure	Type of Cancer Treated with PSs	Maximum Absorption Wavelength (nm)	Extinction Coefficient (M^−1^ cm^−1^)	Ref.
Porfimer sodium	Photofrin	Porphyrin	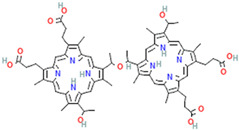	Esophageal, endobronchial, and lung cancer	630	3000	[4,18,40]
5-Amino levulinic acid (ALA)	Ameluz\Levulan	Porphyrin precursor	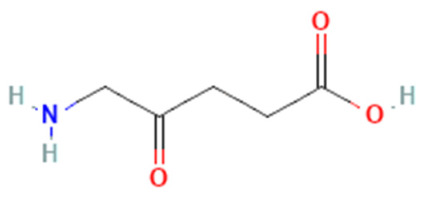	Basal cell carcinoma, squamous cell carcinoma, Actinic keratosis, and head and neck cancer	635	<1000	[4,18,40]
Methyl 5-Amino Levulinic acid (MLA)	Metvix\Metvixia	Porphyrin precursor	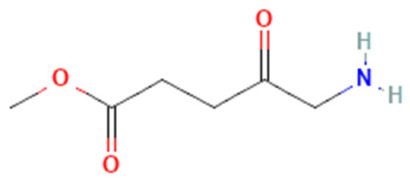	Basal cell carcinoma, Actinic keratosis, Bowen disease, and viral warts	635	<1000	[4,6]
Temoporfin (meta-tetrahydroxy dioxy phenyl chlorine (Mthpc)	Foscan	Chlorin	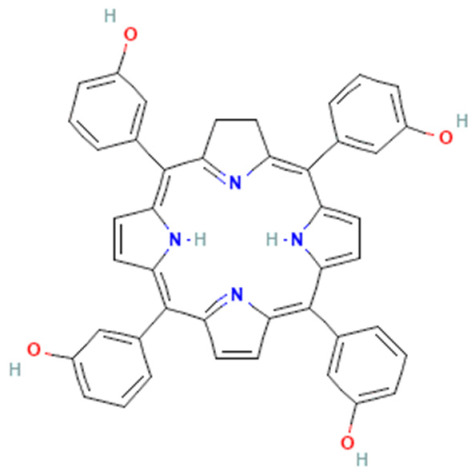	Head and neck, squamous cell carcinoma, and prostate and pancreatic tumors	653	30,000	[6]
Benzoporphyrin derivative mono acid (BPO-MA)	Visudyne\Vetprofin	Chlorin	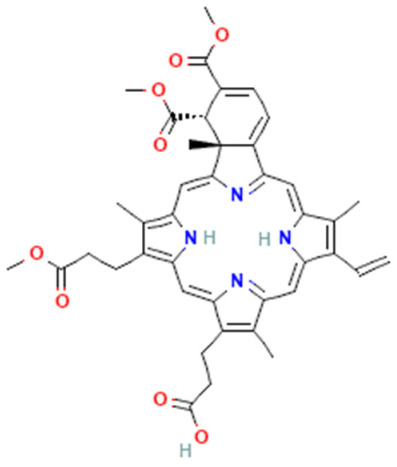	Age-related degeneration and nonmelanoma skin cancer	693	35,000	[6,40]

**Table 3:** Encapsulation of PSs into silica nanoparticles in cancer photodynamic therapy.

Nanocarrier System	Preparation Method of NPs	Size of NPs (nm)	The PS	PS Position	Surface Decoration	Type of Light Source	Power of Light Dose	Irradiation Dose	In Vivo/ In Vitro	Type of Cancer Cells	Outcome	Ref.
Mesoporous silica nanoparticles (MSNs)	-	130	porphyrin	Encapsulated	Dimannoside-carboxylate grafted on the surface of Np	Laser	0.003	650	In vitro	Prostate adenocarcinoma (LNCaP)	The development of new ligands with a better affinity for the receptor on cancer cells increases cell death and reduces the incubation time.	[63]
Periodic mesoporous organosilica nanoparticles (PMOs)	Sol–gel method	447	Tetrasiylated porphyrins	On surface	Loaded with gemcitabine hydrochloride	Laser	2	650	In vitro	Breast cancer cells (MCF-7)	Two-photon excited PDT combined with gemcitabine delivery led to synergy and a very effective cancer cell killing.	[64]
Mesoporous silica nanoparticles (MSNs)	Oil−water biphase stratification	88	TMPyP	Encapsulated	Capped with gold nanoparticles and PEG	Laser	0.3	655	In vivo	Breast cancer cells (MCF-7)	In vitro, cytotoxicity assay has shown that the viability of MCF-7 cells treated with nanophotosensitizer under irradiation reaches the lowest value of 30%.	[65]
Periodic mesoporous organosilica nanoparticles (PMOs)	-	106	Protoporphyrin IX (PpIX)	Encapsulated	-	Green laser light	18	532	In vitro	Colon carcinoma (HT-29)	Showed a significant PDT effect on colon carcinoma and Gram-negative bacteria.	[66]
Silica nanoparticles	Sol–gel method	80	porphyrin	Encapsulated	-	Laser	0.1	635	-	-	The photoactive porphyrin silica nanocomposite particles can generate lethal singlet oxygen.	[67]
Gold nanorods (AuNR)	Stöber method	180	4-carboxyphenyl porphyrin	On surface	Coated with SiO_2_	Far-red radiation	0.5	660	In vivo	Lung carcinoma epithelial cells (A549)	The prepared nanosystem possesses good biocompatibility and can efficiently generate singlet oxygen.	[68]
Rod-like mesoporous silica NPs (MSNR)	Co-condensation process	210	TPPS4	On surface	Coated with gold nanoshell modified with ultrasmall gadolinium (Gd)	Laser	1	660	In vitro and in vivo	Murine breast cancer cells (4T1)	The nanocomposite exhibited high photothermal conversion efficiency and superior ROS productivity.	[69]
Mesoporous silica nanoparticles (MSNP)	Aqueous phase regrowth method	79.5 ± 5.2	Zn-phthalocyanine	Encapsulated	The surface modified with PEG and loaded with cetuximab	Red-light laser	0.05	685	In vitro and in vivo	Pancreatic cancer cell lines (PANC-1, ASPC1, MIA-PACA-2)	In vitro cell uptake experiment showed high uptake into cancer cells. For in vivo studies, the system revealed that the ratio of necrosis in tumor tissue was higher than in untreated cells.	[70]
Mesoporous silica nanoparticles (MSNP)	-	164	Zinc (II) phthalocyanine	Encapsulated	-	Red light	0.001	610	In vivo	Hela cells	Serves as a promising nanoplatform for cancer diagnosis and treated PDT.	[71]
Mesoporous silica nanoparticles (MSNP)	-	303 ± 26	Zinc (II) phthalocyanine	Encapsulated	The surface was decorated with PEG and loaded with cetuximab	Red light	0.009	630	In vitro	Pancreatic cancer cells	Imidazole-capped cetuximab targeted MSNP are excellent vehicles for selective delivery of ZnPcOBP to pancreatic cancer cells expressing the EGFR factor.	[72]
Silicon nanomicelle	-	<10	phthalocyanine	On surface	-	Laser	0.2	671	In vitro and in vivo	4T1 murine breast cancer cells	Tumor cells treated with phthalocyanine silica nanomicelle in the presence of light lead to complete elimination with recurrence, which is greater than ordinary PS without PH response.	[73]
Mesoporous silica-coated upconversion nanoplatform	Aqueous phase regrowth method	-	Silicon phthalocyanine dihydroxide (SPCD)	Encapsulated	Loaded with Au nanoparticles on the surface, and a small molecule DC50 was loaded into the silica shell	NIR	2	808	In vitro and in vivo	Prostate cancer cells (H1229)	The nanoplatform exhibited specific cytotoxicity that was based on the expression levels of Atox1 and CCS protein and promoted the selective accumulation of ROS in particular tumor cells.	[74]
Upconversion nanoparticles (UCNP)	-	125.8 ± 44.04	Zinc phthalocyanine (ZnPc) and merocyanine 540 (MC540)	On surface	Coated with mesoporous silica NPs and surface decorated with gold NPs	NIR	1	980	In vitro	Prostate cancer cells (PC 3)	Successfully produced an efficient nanoplatform MC540/ZnPc-UCNP@Au for superficial and deep-seated PC 3 cells.	[75]
Amphiphilic copolymer	Nucleophilic substitution process	100	Benzyl ester dendrimer silicon (D-Si) and phthalocyanine (Pc)	Encapsulated	-	Laser irradiation	1	670	In vitro	Glioma cells (U251)	The cell viability of glioma cells was decreased to only about 26% after treatment.	[76]
DSPE-PEG_2000_	Co-precipitation method	95	Cholesterol silicon (IV) phthalocyanine (Chol-Pc)	Encapsulated	-	Two-photon laser images	-	730	In vitro	Breast cancer cells (MCF 7)	The nanophotosensitizer effectively mediated photodynamic therapy to kill the breast cancer cell.	[77]
Hollow mesoporous silica nanoparticles (HMSNs)	Stöber method	120	Chlorin e6 (Ce6)	On surface	Loaded with metformin and catalase	Laser	0.5	660	In vitro and in vivo	Breast cancer cells (Bcap37)	The synthesized nonintelligent response system effectively inhibited tumor growth and provided a possibility for tumor imaging diagnosis.	[80]
Mesoporous silica nanoparticles (MSNs)	Stöber method	175	Chlorin e6 (Ce6)	On surface	Decorated with gadolium (Gd) complex and PEG, and also with DOX	Laser	0.5	660	In vivo	Murine squamous cell carcinoma (SCC 7)	The nanophotosensitizer is considered a promising drug carrier for MR imaging-guided photodynamic chemotherapy of cancer.	[81]
Mesoporous silica nanoparticles (MSNs)	-	100	Chlorin e6 (Ce6)	Encapsulated	Platinum nanoparticles immobilized onto channel and TPP decorated on the surface of the 3D-dendritic MSNs	Laser	0.05	660	In vitro	Lung cancer cells (A549)	Enhanced the PDT effect of killing A549 cells and promoted a new hydrogen peroxide activatable strategy to overcome hypoxia of tumor cells.	[82]
Mesoporous silica nanorods (MSNRs)	-	200	Chlorin e6 (Ce6)	Encapsulated	Capped with PEG and gold nanoparticles	Laser	1	660	In vitro and in vivo	Murine breast cancer cells (4T1)	This nanocarrier can serve as a drug delivery platform with high drug loading capacity and enhance the cellular uptake efficiency.	[83]
Mesoporous silica nanoparticles	-	-	Chlorin e6 (Ce6)	Encapsulated and on surface	The triphenylphosphonium was anchored on the NPs	Laser	0.1	655	In vitro and in vivo	Murine breast cancer cells (4T1)	The nanophotosensitizer can generate a large amount of ROS in mitochondria, which cause dysfunction and cell apoptosis.	[85]
Periodic mesoporous organosilica nanoparticles (PMO)	-	105 ± 12	Chlorin e6 (Ce6)	Encapsulated	Loaded with Prussian blue nanoparticles	Laser	1	660	In vivo	Glioma cell lines (U87MG)	The histopathology analysis demonstrates that this oxygen-evolving nanoplatform can effectively elevate singlet oxygen to inhibit tumor growth without obvious damage to major organs.	[85]
Mesoporous silica nanoparticles (MSNs)	-	-	Chlorin e6 (Ce6)	Encapsulated	Loaded with DOX and decorated with HA	Laser	0.05	670	In vitro	Murine squamous cell carcinoma (SCC 7)	Demonstrated that HA-MSNs are favorable nanocarriers with a remarkable CD44 (receptor on cancer cells)-targeting capability for effective dual drug delivery.	[86]
Mesoporous silica nanoparticles (MSNs)	-	313 ± 21	Chlorin e6 (Ce6)	Encapsulated	-	Diode laser	0.1	665	In vitro and in vivo	Breast and glioma cancer cells	Conjugation of Ce6 to MSNs significantly enhanced the cellular uptake and PDT response of Ce6 for both in vitro and in vivo levels.	[87]
Hollow mesoporous silica nanoparticles	-	150	Chlorin e6 (Ce6)	Encapsulated	Serum albumin-integrated manganese dioxide nanoparticles (BSA-MnO_2_) were anchored on the surface and loaded with DOX	Laser	1	660	In vitro and in vivo	Cervical carcinoma	The in vitro and in vivo experiments have confirmed that the nanoplatforms effectively suppress human cervical carcinoma via synergistic therapy.	[88]
Silica nanoparticles (SiO_2_)	-	-	Chlorin e6 (Ce6) and manganese oxide (MnO_x_)	Encapsulated	Coating with mesoporous SiO_2_ and capped with PEG	Laser	0.05	660	In vitro and in vivo	Murine breast cancer cells (4T1)	Both in vivo and in vitro results confirm that the nanosystem can be applied to MRI-guided sufficient PDT with reduced side effects.	[89]
Silica nanoparticles (SLN)	Water in oil microemulsion	115.6 ± 1.6	Chlorin e6 (Ce6)	Encapsulated	Decorated with cell membrane derived from SGC7901 cells (CM)	NIR light	1	680	In vitro and in vivo	Colon cancer cells (HT 29)	Demonstrated that CM/SLM/Ce6 showed a better anticancer outcome compared to SLN/Ce6.	[90]
Upconversion nanoparticles (UCNPs)	-	85.63	Chlorin e6 (Ce6)	On surface	Coated with mesoporous silica and loaded with DOX	NIR	-	980	In vitro	Murine squamous cell carcinoma (SCC 7)	UCNPs@msSiO_2_-DOX/Ce6 decreased the SCC7 cell viability and provided a dual function of drug delivery and generation of ROS.	[91]
Fe_3_O_4_ nanoparticles	Sol–gel method	162 ± 11.3	Chlorin e6 (Ce6)	On surface	A mesoporous silica shell surrounded the nanoparticles core. DOX was loaded into mSiO_2_ and a polydopamine coating layer. Finally, HAS molecules were conjugated to the polydopamine surface	Red light	0.005	660	In vivo	Glioma tumor cells	Fe_3_O_4_@mSiO_2_/DOX@HSA Ce6 nanoplatform was guided to the tumor region by magnetic targeting, and this nanoplatform suppressed glioma tumor growth efficiently.	[92]
Upconversion nanoparticles (UCNPs)	-	-	Chlorin e6 (Ce6)	Encapsulated	Coated with mSiO_2_ and loaded with antibody glypican 3 on the surface	NIR	2	808	In vivo and in vitro	Liver cancer	The in vivo and in vitro studies demonstrate that this nanosystem is safe and has a potential therapeutic option for liver cancer.	
Mesoporous organosilica nanoparticles	Sol–gel method	120	Indocyanine green (ICG)	Encapsulated	Encapsulated with macromolecular catalyst	Laser	0.8	808	In vitro and in vivo	Murine breast cancer cells (4T1)	The obtained MONs could serve as an intelligent nanothermometer agent for photoacoustic/ ultrasound dual-modality imaging-guided tumor PDT.	[95]
Hollow mesoporous silica nanoparticles (HMSNs)	The Stöber method	269	Indocyanine green (ICG)	Encapsulated	Dopamine-modified hyaluronic acid adhered to the surface of the inorganic nanoparticle and loaded with DOX	NIR	2	808	In vitro	Murine breast cancer cells (4T1)	In vitro cell experiments perfectly showed that ID@HMSN-HA could inhibit murine mammary carcinoma cell via chemotherapeutic combined with photodynamic therapy.	[96]
Mesoporous silica nanoparticles	-	193.4	Indocyanine green (ICG)	Encapsulated	Cored with PFE and decorated with PEG and FA on the surface of nanoparticles	NIR	1.5	808	In vitro and in vivo	Breast cancer cells (MCF 7)	The formed nanophotosensitizer exhibited superior antitumor efficiency.	[97]
Hollow mesoporous silica nanoparticles	Facile method	250.5 ± 8.1	Indocyanine green (ICG)	Encapsulated	Loaded with l-methanal (LM) and DOX and the surface decorated with TPP	NIR	0.8	808	In vitro	Breast cancer cells (MCF 7, A549)	The remarkable synergistic combination of DOX-based chemotherapy and ICG-mediated phototherapy in this versatile nanoplatform, which offers a compelling strategy for cancer treatment.	[98]
Mesoporous silica nanoparticles	Sol–gel method	201.1 ± 17.3	Indocyanine green (ICG)	On surface	Decorated with TPP and with α- tocopherol succinate on the surface	Laser	0.5	808	In vivo	Breast cancer cells (MCF 7)	Reduce innate oxygen consumption by blocking mitochondrial ROS burst in PDT.	[99]
Gold nanorods (AuNR)	-	-	Indocyanine green (ICG)	Encapsulated	Mesoporous silica	NIR	0.1	808	-	Liver cancer cells (Hep-G2)	The optimal configuration activated almost twice the temperature increase, five times the reactive oxygen species generation, and finally three times the cancer cell killing ability compared to free ICG.	[100]
Dendritic large pore mesoporous silicon Nps (DLMSNs)	Dual template sol–gel method	153.9 ± 12.6	IR 780	Encapsulated	Co-loaded with DOX and camouflaged with LPHM	NIR	2	808	In vivo	Murine breast cancer cells (4T1) and (TNBC)	LPHM@DDI nanoparticles exhibited synergistic cytotoxic and apoptosis-induced activity in cancer cells and suppressed tumor growth.	[101]
Semiconductor polymer dots hybrid mesoporous silica nanoparticles	Improved emulsion solvent evaporation method	152.9 ± 9.41	Triapazamine (Tpz)	Encapsulated	Treated with polyethylene glycol and folic acid	NIR	1	808	In vitro and in vivo	-	Local hypoxia caused by molecular oxygen consumption simultaneously activates the cytotoxicity of Tpz, which effectively kills activated macrophage and inhibits the progression of arthritis.	[102]
Mesoporous silica nanoparticles	-	-	Verteporfin (Ver)	Encapsulated	-	Red light	-	693	In vitro and in vivo	Skin melanoma cancer cells	The red-light irradiated Ver-MSNs can significantly reduce tumor angiogenesis of skin melanoma.	[103]
Hollow mesoporous silica NPs (HMSN)	The Stöber method	120 ± 10	Rose Bengal (RB)	Encapsulated	Hyaluronic acid (HA) was modified on the surface and loaded	Uv light	0.01	532	In vitro	Murine mammary cancer cells (4T1) and (TNBC)	Could precisely target murine mammary cells and effectively inhibit tumor cell viability with chem-photodynamic synergistic therapy.	[105]
Mesoporous silica nanoparticles (MSNs)	-	197	curcumin	Encapsulated	Capped with PEG	-	0.02	430	In vitro	Hela cells	MSN-PEG@Cur could be effectively endocytosed into cells and release Cur, which can promptly generate ROS upon irradiation.	[106]
Mesoporous silica nanoparticles (MSNs)	-	199 ± 24	Ru (II) polypyridine complex	Encapsulated	Functionalized with folic acid	-	-	480 or 540	In vivo	Lung and ovarian cancer cells	The conjugates were found to be nontoxic in noncancerous human normal lung fibroblast cells, while showing a phototoxic effect upon irradiation in ovarian cancer cells.	[107]
Mesoporous silica nanoparticles (MSNs)	Sol–gel method	72.5 ± 4.8	Methylene blue (MB0	Encapsulated	Capped with PEG and loaded with DOX	Laser	0.015	660	In vivo	Murine breast cancer cells (4T1)	Chemo-photodynamic therapy elicited long-term systemic antitumor immunity for suppressing distant and metastatic tumor growth as well as inhibiting tumor recurrence.	[108]
Mesoporous silica nanoparticles (MSiO_2_)	-	140	Manganese dioxide nanosheets	On surface	A great amount of gold nanoclusters were loaded onto the surface	Laser	0.1	635	In vivo	Breast cancer cells (MDA-MB-435)	The hydrogen peroxide response of nanophotosensitizer showed excellent off/on modulation and enhancement of magnetic resonance imaging and PDT and was a promising intelligent nanoprobe for safe and high-efficiency theranostic outcomes.	[109]
Mesoporous silica nanoparticles	-	155	Gold nanoparticles	On surface	Loaded with DOX and decorated with PEG	-	0.04	550	In vitro	Hela cells	In vitro data indicate that the loaded DOX could be controlled released from the formed nanophotosensitizer and could generate ROS-induced apoptosis of tumor cells.	[110]
Mesoporous silica nanoparticles (MSNs)	-	-	Singlet oxygen	On surface	Integrated with a nitric oxide photodoner and encapsulated with DOX	Green light	-	532	In vitro	Melanoma cancer cell line (A375)	Preliminary biological results performed using A 357cancer cells show good tolerability of the functionalized MSNs in the dark and potential activity of DOX upon radiation.	[111]
Hollow mesoporous silica nanoparticles	Optimized selective etching method	115	Pheophorbide (PA)	Encapsulated	Loaded with DOX. Capped and crosslinked with chitosan and glycidoxypropyl- trimethoxy silane and targeted with folic acid	Laser	0.5	680	In vitro and in vivo	Oral squamous cell carcinoma (KB cells)	The nanocarrier showed excellent drug-controlled release properties based on the pH-dependent swelling effect of the coating layer.	[112]
Mesoporous silica nanoparticles (MSNP)	-	100	BODIPY	On surface	Decorated the surface using PEG	Green light	0.016	500	In vitro	Hela cells	The formed nanophotosensitizer has good toxicity against tumor cells, and adding glutathione could further improve the photodynamic effect.	[113]
Mesoporous silica nanoparticles (MSNs)	-	50 ± 10	BODIPY dyes	On surface	Functionalized with PEG and folic acid (FA)	Red light	-	518	In vitro	Hela cells	Photosensitizer silica NPs functionalized with PEG and FA proved to be suitable and biocompatible nanosystem, able to overcome the drawbacks of free PS.	[114]
Upconversion nanoparticles	Sol–gel reaction	<100	Mercy anine 540 (MC 540)	Encapsulated	Decorated with tumor antigens (TF) and a tumor cell fragment and loaded with chicken oval albumin protein (OVA), and the surface coated with mesoporous silica nanoparticles	NIR	0.5	980	In vivo	Colon cancer	The formed nanoparticles more effectively inhibited tumor growth and showed the best synergistic immunopotentiation.	[115]
Silica nanoparticles (SNPs)	Water in oil microemulsion procedure	60	Hexamolybdenum	On surface	Decorated with 3500 amino groups	Light-emitting diode	0.03	420	In vitro	Breast cancer cells (MCF 7)	The prepared C-SNs hybrid reveals a significant photodynamic therapy effect on the breast cancer cell line.	[116]
Silica nanoparticles	Reverse micellar method	-	Cichorium pumilum (CP)	Encapsulated	-	-	0.27	350	In vitro	Osteosarcoma cells line (U-2)	The engineered silica nanoparticles loaded with photosensitizer enhance the PDT by increasing CP bioavailability.	[117]
Silicon nanoparticles SiNPs	-	-	ruthenium (Ru)	On surface	Decorated with folic acid	Laser	-	655	In vitro and in vivo	Hela cells	Serve as a targeted two-photon fluorescence imaging probe and kill cancer cells via PDT in vitro.	[118]
Silica nanoparticles	-	78.43 ± 19.92	Xylan carrying TPPOH	On surface	Capped with (3-aminopropyl) triethoxysilane (APTES)	Red light	-	630–660	In vitro	Colon cancer (HCT116 and HT-29 cells	In vitro analysis showed that the nanophotosensitizer is more effective than free TPPOH.	[119]
Mesoporous silica ruthenium nanoparticles (MRN)	Double template method	138 ± 8.1	[Ru(bpy)_2_ (tip)]^2^ (RBT)	Encapsulated	Transferrin (TF) and aptamer AS1411 are grafted on the surface of MRN with high loading capacity	Laser	0.25	808	In vitro and in vivo	Glioma cancer cells	Effective treatment of brain tumor.	[120]

## Data Availability

The data presented in this study are available in this article.
